# Characterization of water microbiota and their relationship with resident oysters during an oyster mortality event

**DOI:** 10.1128/spectrum.02881-23

**Published:** 2024-08-20

**Authors:** Mingkun Liu, Qingyuan Li, Wenwen Xu, Luping Wang, Fucun Wu, Lintao Tan, Li Li, Guofan Zhang

**Affiliations:** 1 CAS and Shandong Province Key Laboratory of Experimental Marine Biology, Center for Ocean Mega-Science, National and Local Joint Engineering Key Laboratory of Ecological Mariculture, Institute of Oceanology, Chinese Academy of Sciences, Qingdao, China; 2 Laboratory for Marine Biology and Biotechnology, Laoshan Laboratory, Qingdao, China; 3 Key Laboratory of Breeding Biotechnology and Sustainable Aquaculture, Chinese Academy of Sciences, Wuhan, China; 4 Shandong Technology Innovation Center of Oyster Seed Industry, Qingdao, China; 5 Southern Marine Science and Engineering Guangdong Laboratory (Zhanjiang), Zhanjiang, China; 6 University of Chinese Academy of Sciences, Beijing, China; 7 Rushan Marine Economy and Development Center, Rushan, China; 8 Laboratory for Marine Fisheries Science and Food Production Processes, Laoshan Laboratory, Qingdao, China; USDA-ARS National Center for Cool and Cold Water Aquaculture, Kearneysville, West Virginia, USA

**Keywords:** environmental microbiota, intestinal microbiota plasticity, oyster mortality, resident and transient microbes, microbial ecology

## Abstract

Microorganisms are vital for the health of marine invertebrates, and their assembly is driven by both deterministic and stochastic factors that regulate residents (innate to the host) and transients (from ambient water). However, the role of water microbiota and the significance of deterministic and stochastic processes in aquatic hosts facing mortality threats are largely unknown. This study examines the shifts in water microbiota during an oyster mortality event using amplicon sequencing and compared with those of resident oysters to disentangle the balance of the deterministic and stochastic factors involved. Water temperature and dissolved oxygen significantly shape the microbial community with a distinct monthly pattern, and *Cyanobacteria* blooms might exacerbate oyster mortality. The comparative analysis of microbial communities in oysters and water revealed that ≤ 21% of the genera were shared between oysters and water, implying that water microbiota cannot easily transfer into oysters. Furthermore, these shared genera had different functions, with oysters more involved in promoting host digestion and nutrient acquisition and water bacteria enriched more in functions promoting their own growth and survival. These findings illustrate that oysters may possess specific selection or barrier mechanisms that permit a small percentage of transients, controlled by stochastic factors and having a minimal effect on oyster mortality, to enter, whereas the majority of oyster microbiota are residents governed by deterministic factors. Consequently, oysters exhibit some plasticity in their symbiotic microbiota, enabling them to maintain microbial homeostasis and adapt to complex microbial surroundings. This may be a shared mechanism among marine invertebrates for survival in complex marine environments.

**IMPORTANCE:**

Pacific oysters are widely cultured and play vital ecological roles. However, the summer mortality hinders sustainable oyster farming. Untangling causative mechanisms of oyster mortality is a complex task due to the intricate “interactome” involving environmental factors, hosts, and pathogens. Interactions between hosts and microorganisms offer an ideal avenue for investigating the truth. We systematically investigated the microbial community in water and resident oysters during a summer mortality event and proposed that the assembly of oyster microbiota is primarily governed by deterministic processes independent of mortality. Pathogens mainly originate from resident members of the oyster microbiota, with a limited influence from the microbial community in the water. Additionally, environmental degraders, such as *Cyanobacteria* blooms, cannot be overlooked as a contributing factor of oyster mortality. This study evaluated the weight of deterministic and stochastic factors in microbial assembly during an oyster mortality event and greatly broadened our understanding of the “interactome” through the interaction between oysters and water in microbiota.

## INTRODUCTION

Microorganisms inhabit a diverse range of habitats and are the key drivers in ecosystems (1, 2). They also participate in various biological functions such as nutrient cycling (3), immune defense (4), and development (5). Microbiome-wide studies have shown that variations in symbiotic microbiota are correlated with various diseases (6). Unlike terrestrial animals, marine animals are exposed and coerced by a plethora of microorganisms in the water and thus exist in a closer association with microbes in the surroundings, where microbiota play an imperative function in defining the fitness of marine animals (7). It is traditionally believed that ambient water functions as a reservoir for microorganisms that constitute the symbiotic microbiota of marine animals (8). Changes in the environmental microbiome, particularly blooms of pathogenic bacteria, can dramatically affect host microbiome assembly and environmental adaptation (9). Disorder or collapse of the host microbiota is often fatal, and a significant change in the host microbial community is regarded as a prefigure of disease or mortality outbreaks in aquatic animals (9, 10). Therefore, it is indispensable to consider ambient microbiota when investigating disease or mortality outbreaks in aquatic animals.

The shift of microbiota from aquatic environments to marine animals is not unrestricted. The assembly of microbial communities in marine organisms is governed by both deterministic and stochastic processes (11). Deterministic factors such as host inheritance, immunology, and metabolism create consistent patterns of microbiome membership that result in persistent resident communities. Stochastic processes such as immigration events can result in random fluctuations in microbiome members that are transient regarding their association with the host (11, 12). Microbial members that are explicitly selected by the host can be considered resident associates, whereas those driven by external factors (e.g., environmental microbiota) can be considered transient associates (11). Disentangling the balance between deterministic and stochastic processes is essential for a deeper understanding of bacteria-related mortality. Determining resident and transient members can provide a valuable reference for managing microbiota-based monitoring strategies.

For aquatic animals, the weights of deterministic and stochastic processes vary depending on the degree of manual intervention. Intensive aquaculture with manual intervention can serve as a moderate and stable environment in which the aquatic animal microbiota tend to change synchronously with the water microflora. For example, during Nile tilapia (*Oreochromis niloticus*) development, alterations in gut bacterial communities correlated with changes in water communities, and the water microbiota could successfully transfer into the fish gut (13). Distinct water treatments between recirculating aquaculture systems and flow-through systems induced differences in Atlantic cod (*Gadus morhua*) larval microbiota (14). No legacy effect in Atlantic cod microbial communities of initial rearing conditions was observed after varying the water treatment system, and the host microbiota changed synchronously with their surroundings (15). Under these circumstances, with little environmental stress, stochastic processes dominate the entry of water transient members into animals. The common cognition that aquatic fish microbiota are significantly influenced by and change with the surrounding microbiota has been widely established and applied to microbial management in fish industrial aquaculture (16).

Nevertheless, a different pattern is widely unveiled in complex and changeable environments, such as semi-artificial ponds and open waters in the wild. Zhao *et al*. (17) found that the gut microbiota of freshwater shrimps (*Macrobrachium nipponense*) cultured in ponds differed significantly from the environmental microbiota. Huang *et al*. (18) reported that Pacific white shrimp (*Litopenaeus vannamei*) bacterial community structures were significantly distinct from ambient water and sediment, exhibiting the lowest *α*-diversity and a small number of shared operational taxonomic units (OTUs). The microbiota divergence between water and its inhabitants is also reflected in bivalves such as mussels (19) and oysters (20, 21). In a highly convincing experiment, Andrea *et al*. (11) maintained Eastern oysters (*Crassostrea virginica*) from different wild populations in a common garden for 6 weeks and found that the oysters maintained distinct microbial communities that reflect their wild population of origin instead of their surroundings. Stevick *et al.* (21) proposed that Eastern oysters could select and amplify certain bacterial species through filtering selection and niche colonization. Thereupon, in the complicated environment of open waters with more stresses, the microbiota community is assembled under the control of deterministic processes, leading to the shellfish microbiota resembling an “island” ecosystem that appears to be mainly shaped by the internal environment and certain other selective changes around the host (12), exhibiting some plasticity to adapt to changes in the water-cultured environment (20, 21). However, the precise understanding of the relationship between deterministic and stochastic processes, as well as the dynamics between resident and transient microbial members, remains largely elusive, especially in situations where diseases or mortality events lead to substantial shifts in the symbiotic microbiota of the host. It is crucial to further investigate whether environmental microbiota, particularly pathogens, gain increased access to aquatic animals during conditions of severe impairment in host functions such as immunology and metabolization.

Pacific oysters (*C. gigas*) are cultured globally and play a significant role in the regulation of water quality and ecological stability. However, recurrent mortality episodes occurring during hot summers have become an irreconcilable obstacle to the healthy development of the oyster farming industry (22). In our previous study, we performed a comparative analysis of the differences in the intestinal microbiota and immune responses between resistant and susceptible oysters that exhibited varying survival rates during mortality events (23). The monthly monitoring results showed that the mortality lasted from August 18 to September 22. The microbiota structure of oysters exhibited a temperature-dependent pattern. Two potential pathogens, Betaproteobacteriales and Acidobacteriales, were significantly enriched in susceptible oysters at the later mortality stage (September). Resistant oysters possessed a stronger ability to inhibit pathogen proliferation owing to their enhanced immunological functions, whereas suppressed immunological functions followed by continuous pathogen proliferation were detected in susceptible oysters. However, it is unclear where these pathogens originated from. Did these bacteria persistently reside in oysters and proliferate under suitable environmental conditions (such as rising temperatures)? Alternatively, the balance between deterministic and stochastic processes is altered (due to ineffective immune functions in susceptible oysters); thus, stochastic factors promote the transfer of pathogens into oysters from the surroundings. In the present study, we characterized the microbiota of ambient waters collected simultaneously with the oyster samples presented in our previous study (23) to 1) investigate the microbial characteristics of aquatic water during an oyster mortality event, 2) compare the microbial composition and predicted functions of oysters and their surroundings, and 3) elucidate the weight of deterministic and stochastic processes in oyster microbiota assembly during a mortality event. This study provides new insights into the balance between deterministic and stochastic forces driving community dynamics in commercially and ecologically important marine species and contributes to a deeper understanding of oyster summer mortality events.

## RESULTS

### Overall characteristics of microbial data

Following quality control, 1,988,414 effective reads were produced from 18 samples across the six groups. The average N50 and N90 lengths were 450 bp and 441 bp, respectively. The average number of operational taxonomic units (OTUs) was 1,603, with a range of 1,029—2,373, and all samples had Good’s coverage indices of 0.9945—0.9991 (Table S1).

**Fig 1:**
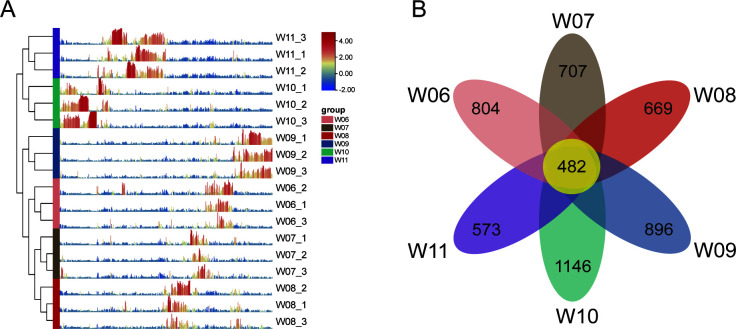
Overall microbiota structure in water samples from June to November. (A) Relative abundance heatmap of all OTUs in all samples. (B) Venn diagram depicting the numbers of shared and unique OTUs among the six groups.

Based on the relative abundance of OTUs, each group clustered independently depending on the sampling date, and each group had exclusively dominant OTUs (Fig. 1). Two distinguishable branches were observed: one containing samples from the high-temperature period (W06, W07, W08, and W09) and the other containing samples from the cold period (W10 and W11). The average water temperature during the high-temperature period (June—September) ranged from 22.8°C to 26.6°C, while during the cold period (October—November), it was 10.5°C to 17.3°C (23). These divergent patterns suggested that temperature played a role in shaping the microbial community in water. Differentiation among the groups was further confirmed by the Venn diagram (Fig. 1B), which revealed that only 6.92% (482) of the OTUs were shared among the six groups. Additionally, 5,277 unique OTUs were observed across the six groups, underscoring their distinct compositions.

### Diversity of the water microflora

**Fig 2:**
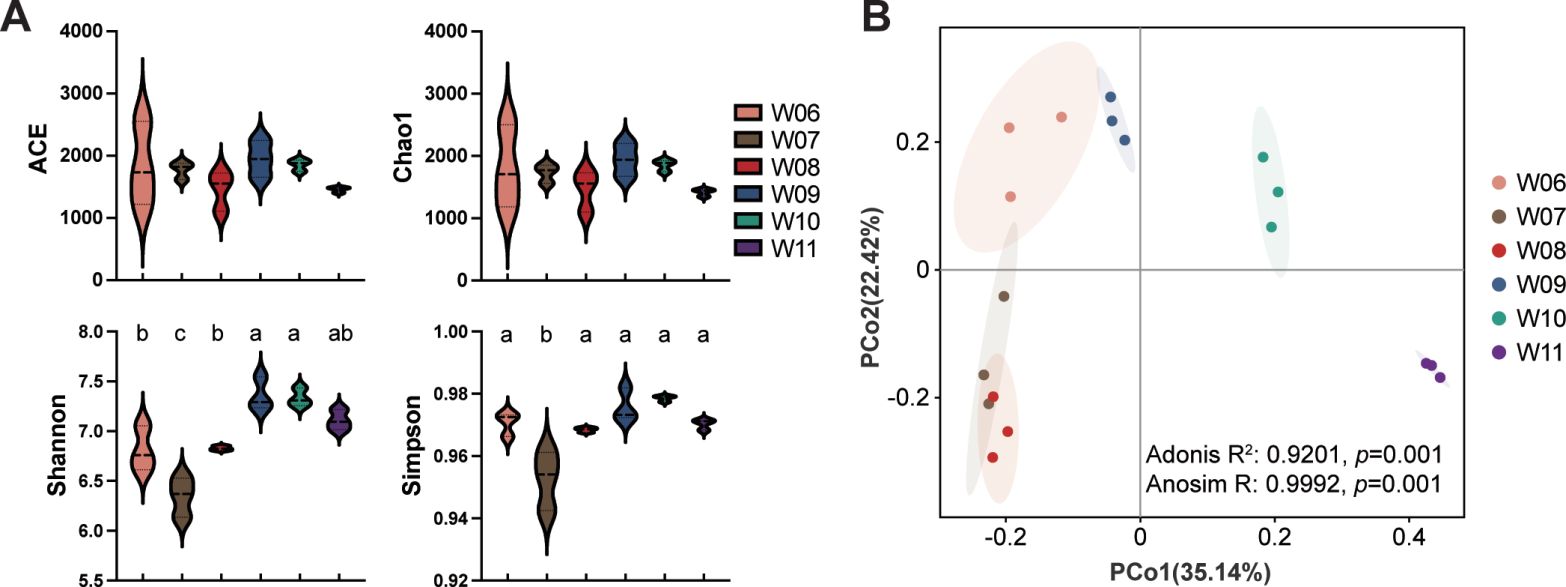
α-diversity (**A**) and *β*-diversity (**B**) of water microbiota. Dissimilar lowercase letters indicate significant differences among groups (*P* < 0.05).

The *α*-diversity indices were calculated and compared among six groups (Fig. 2A). There were no significant differences in the richness indices of ACE and Chao1 among the groups. However, differences were observed in the Shannon and Simpson diversity indices, with low values during warm months and high values during cold months. The analysis of variance (ANOVA) revealed that water temperature and dissolved oxygen significantly affected the Shannon and Simpson diversity indices (Table 1). The principal coordinate analysis (PCoA) plot showed significant segregation of microbiota structure depending on the groups, which was further confirmed via Adonis (R^2^ = 0.9201, *P* = 0.001) and Anosim (R = 0.9992, *P* = 0.001) (Fig. 2B).

**TABLE 1:** ANOVA summary of the effects of environmental factors on *α*-diversity indices^*a*^

		T	Do	Sal	Chl
	Df	1	1	1	1
	MS	59891	161297	436969	2255
ACE	F	0.577	1.555	4.213	0.022
	*p*	0.4609	0.2344	0.0608	0.885
	MS	68346	205305	386209	8471
Chao1	*F*	0.678	2.035	3.829	0.084
	*p*	0.4253	0.1773	0.0722	0.7765
	MS	0.516	1.4304	0.0231	0.0338
Shannon	*F*	13.927	38.609	0.624	0.911
	*p*	**0.00251**	**3.15E-05**	0.4439	0.35726
	MS	0.0001786	0.0005495	0.0000151	0.0000896
Simpson	*F*	3.444	10.597	0.291	1,728
	*p*	**0.04629**	**0.00626**	0.59862	0.21143

^*a*^ Bold *P* values indicate significant differences (*P* < 0.05), as determined by ANOVA. The values of T (temperature), Do (dissolved oxygen), and Sal (salinity) corresponded to those observed in our previous study (23), and the Chl (chlorophyll a) values are given in Table S2.

### Community structure of the water microflora

**Fig 3:**
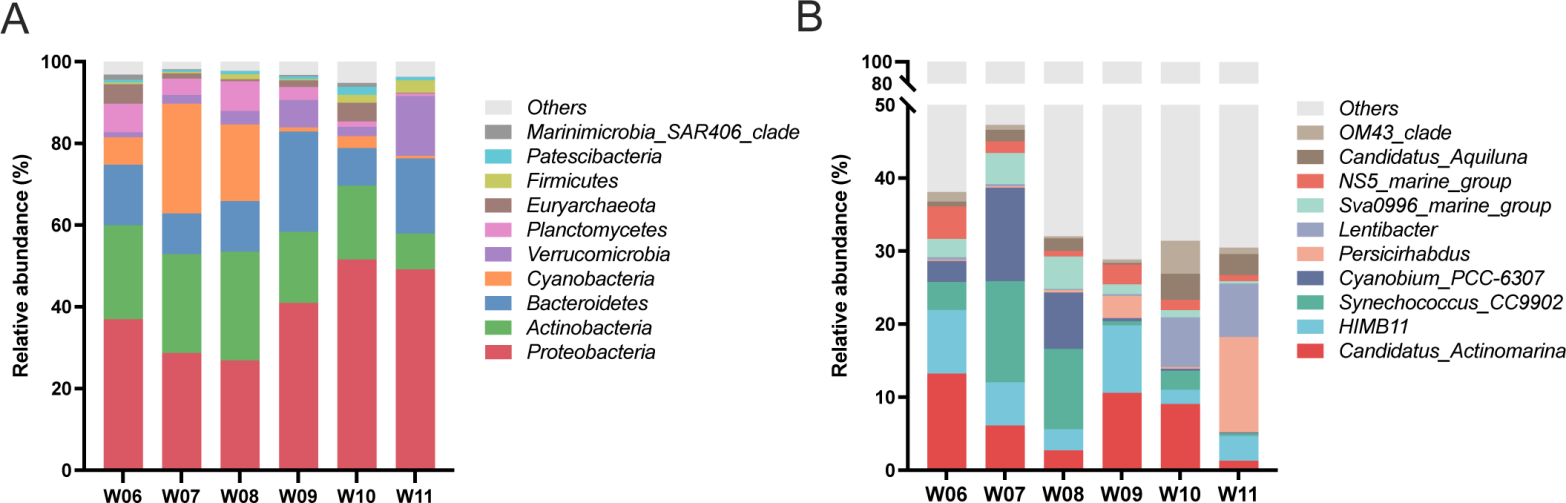
Composition of the water microbiota from June to November at the phylum (**A**) and genus levels (**B**). The top10 categories were shown in the figure, and the rest was indicated as “Others.”

Taxonomic classification revealed that more than 97% of the identified species were bacteria. Proteobacteria, Actinobacteria, Bacteroidetes, Cyanobacteria, and Verrucomicrobia were the dominant phyla ound in the water microbiota (Fig. 3A). Cyanobacteria was highly abundant in the warmer months of W07 and W08, whereas Verrucomicrobia was enriched in the colder month of W11. Wide variations were also detected at the genus level. For example, *Synechococcus_CC9902* and *Cyanobium_PCC-6307* were the dominant genera in W07 and accounted for 56.3% of all identified genera. *Persicirhabdus* and *Lentibacter* were dominant in W11, accounting for 66.8% of the identified genera (Fig. 3B).

**Fig 4:**
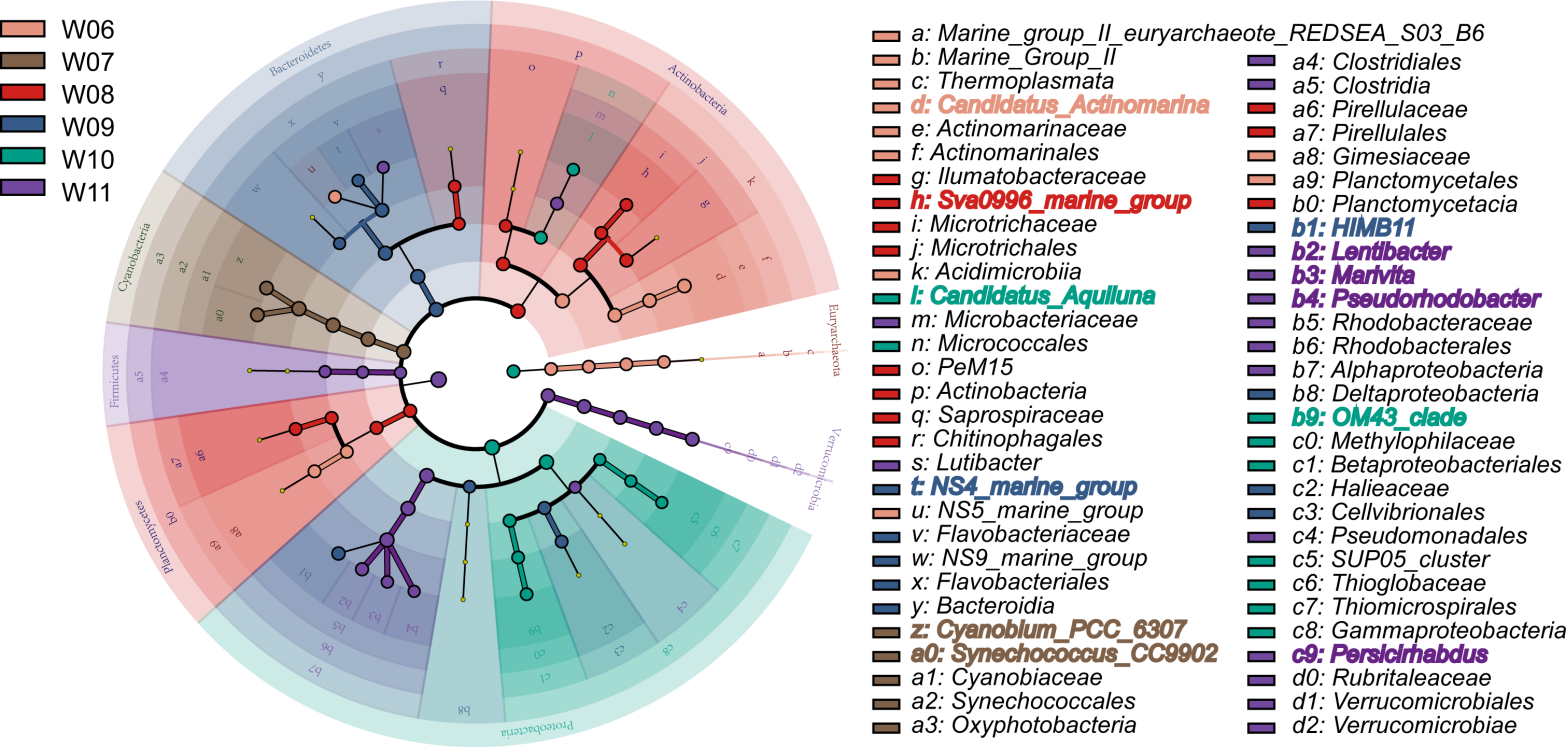
Differentially displayed microbial taxa among six groups based on the Linear discriminant analysis Effect Size (LEfSe) analysis with LDA >4.0 as the threshold.

Linear discriminant analysis Effect Size (LEfSe) analysis identified 59 biomarker taxa with a linear discriminant analysis (LDA) score of >4 across the six groups (Fig. 4). At the phylum level, Cyanobacteria and Verrucomicrobia were enriched at W07 and W11, respectively. At the genus level, 12 taxa were identified as the most discriminative biomarkers distributed across the six groups: *Candidatus_Actinomarina* in W06; *Cyanobium_PCC_6307* and *Synechococcus_CC9902* in W07; *Sva0996_marine_group* in W08; *NS4_marine_group* and *HIMB11* in W09; Candidatus_*Aquiluna* and *OM43*_*clade* in W10; and *Lentibacter*, *Marivita*, *Pseudorhodobacter,* and *Persicirhabdus* in W11. Furthermore, nine of the top 10 genera were included in the biomarker taxa.

### Correlation between water microbiota and environmental factors

**Fig 5:**
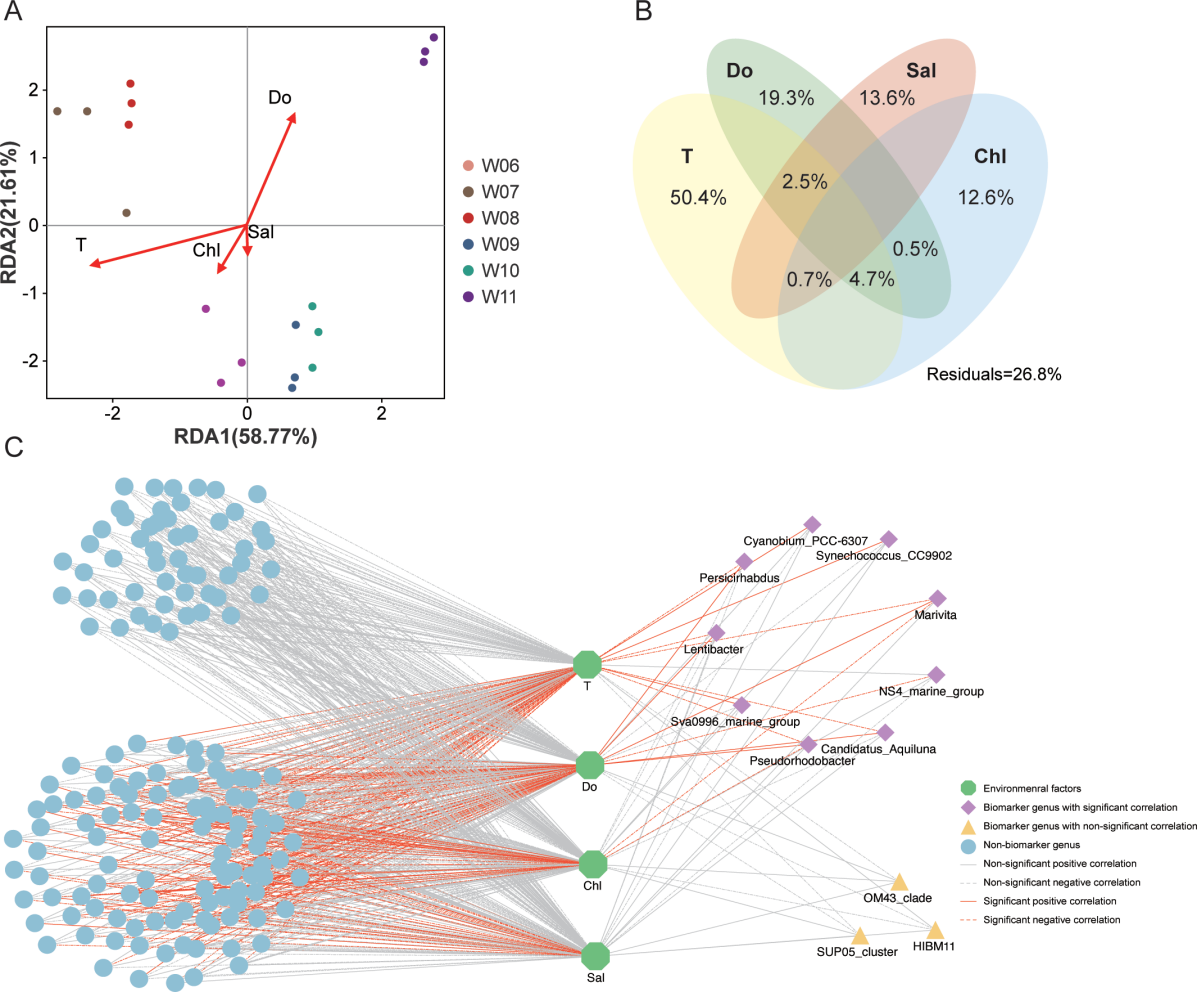
Relationship between water microbiota and environmental factors. Redundancy analysis (RDA) (**A**) and variation portion analysis (VPA) (**B**) between environmental factors and water microbiota at the OTU level. C: Correlation network between environmental factors and water microbial communities at the genus level.

Redundancy analysis (RDA) was conducted to assess the correlation between water microbiota and monitored environmental conditions. The eigenvalues of the first two components explained over 80% of the variation among the groups, whether at the OTU (Fig. 5A) or genus level (Fig. S1A). The results revealed that T and Do are important factors influencing the microbial community in water. Variation portion analysis (VPA) was performed to quantify the contributions of environmental factors to the aquatic microbial community. The complete set of environmental variables collectively accounted for more than 70% of the variation in the water microbial communities. Specifically, at the OTU level, T and Do contributed 50.4% and 19.3% of the microbial variation, respectively (Fig. 5B). At the genus level, these two factors contributed to 49.7% and 20.4% of the variation, respectively (Fig. S1B).

The correlation network revealed that 67% of the identified genera were significantly correlated with environmental factors. Among these genera, 66%, 56%, 30%, and 29% were significantly correlated with T, Do, Chl, and Sal, respectively (Fig. 5C). Focusing on the 12 screened biomarkers at the genus level (Fig. 4), eight genera showed a significant correlation with T: three were positively correlated and five were negatively correlated. Six genera showed a significant correlation with Do: five were positively correlated and one was negatively correlated. Two genera were significantly negatively correlated with the Chl. None of the biomarkers showed a significant correlation with Sal.

### Differences in microbial composition and function between water and oysters

**Fig 6:**
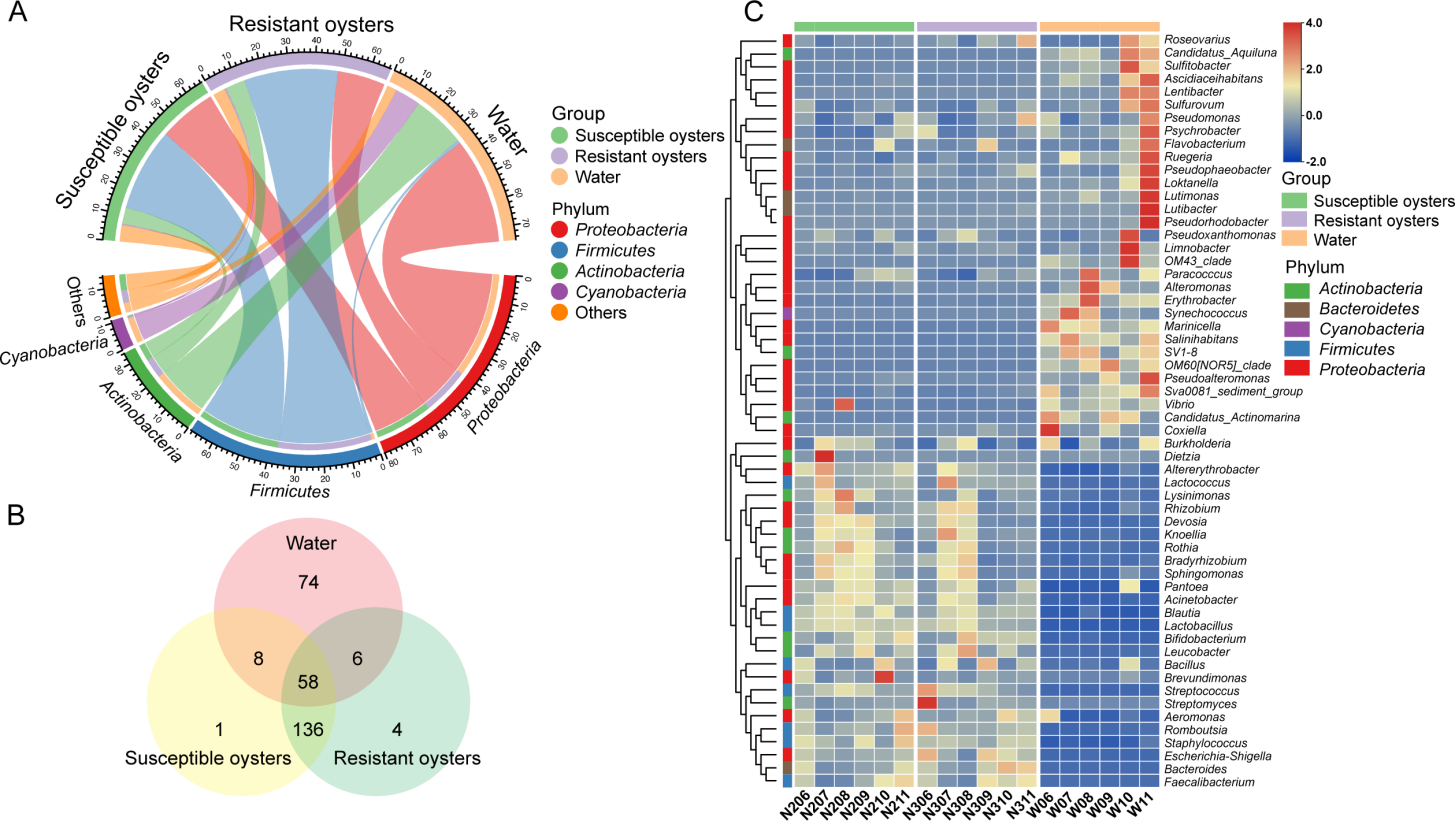
The divergence of microbial composition between water and oysters. (A) Circos plot showing features of dominant phyla in water and oysters. (B) Venn diagram analysis depicting the number of shared and unique genera between water and oysters. (C) Heatmap based on the relative abundance of shared genera between water and oysters.

Microbial communities in water and oysters (23) were re-analyzed and compared. As shown in Fig. 6A, there were marked differences in the dominant phyla between the water and oysters. The abundance of Proteobacteria and Actinobacteria was higher in water than in oysters. Cyanobacteria accounted for 9.48% of the microbial community in water, but less than 1% was detected in the oysters. Conversely, Firmicutes was much more abundant in oysters (over 33%) than in water (only 1.2%). A similar divergence pattern was also observed in the other top 10 phyla (Fig. S2).

At the genus level, the number of identified genera in oysters was 1.5 times that in water, and 91% of the genera in oysters were shared between susceptible and resistant oysters (Fig. 6B). Only 58 genera, accounting for <21% of all identified genera, were shared between water and oysters (Fig. 6B). Of these 58 shared genera, 31 were more abundant in water, whereas 27 displayed the opposite pattern, with a higher abundance in oysters (Fig. 6C). Most genera enriched in water were subordinate to Proteobacteria and Bacteroidetes, whereas a greater number of genera belonging to Firmicutes were found in oysters. Notably, the common pathogens or unfavorable bacteria such as *Vibrio*, *Coxiella*, and *Synechococcus* were less abundant in oysters than in water (Fig. 6C).

**Fig 7:**
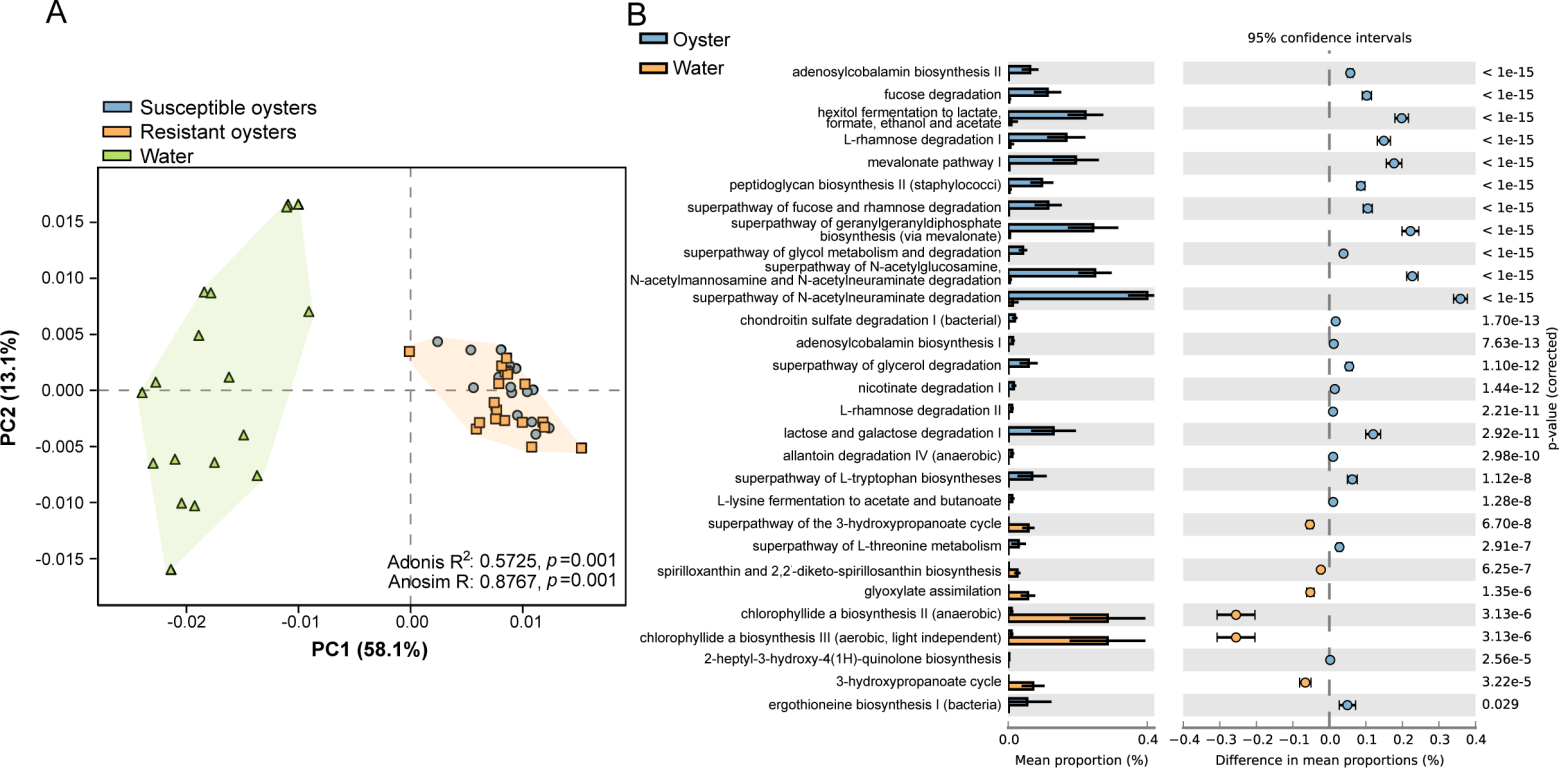
Significant difference in microbial functions between water and oysters based on PICRUSt2 and STAMP analysis. (A) PCA plot of the microflora among the water, susceptible oysters, and resistant oysters. (B) Functional pathways compared between oysters and water.

Functions of the shared microbiota were predicted using PICRUSt2 analysis (Fig. 7). The PCA plot showed significant differences in the enriched functions between water and oysters, which were verified using Adonis (R^2^ = 0.5725, *P* = 0.001) and Anosim (R = 0.8767, *P* = 0.001) (Fig. 7A). Compared with water, most pathways associated with catabolic functions were enriched in oysters, such as fucose and rhamnose degradation, hexitol and L-lysine fermentation, glycol metabolism and degradation, glycerol degradation, and lactose and galactose degradation. A small number of biosynthesis-associated pathways were detected, including adenosylcobalamin, peptidoglycan, and L-tryptophan biosynthesis. Conversely, in water, the enriched pathways mainly involved assimilation and biosynthetic functions, such as spirilloxanthin biosynthesis, glyoxylate assimilation, and chlorophyllide a biosynthesis (Fig. 7B).

## DISCUSSION

As the understanding deepens, the outbreak of oyster mortality events has evolved from a “classical view” (one pathogen one disease) to an “ecological view,” in which multiple factors contribute to or amplify the mortality process (24). It is now widely accepted that oyster mortality events result from interactions among the host, environmental factors, and pathogens (22). Due to the complexity of this “interactome,” it is challenging to untangle the causative mechanisms of massive mortality. Our previous study elucidated the interactions between host immunity and intestinal microbiota and found that high temperatures promoted the proliferation of unfavorable bacteria coupled with suppressed immunological functions, leading to a high mortality rate in susceptible oysters (23). In this study, we ulteriorly extended our understanding of the “interactome” to include the environmental microbiota to clarify the role of water microflora during a mortality event and their relationship with oysters. These results not only improve our cognization of oyster summer mortality but also provide novel insights into the assembly mechanism of oyster microbiota.

### Outbreak of *Cyanobacteria* exacerbated the oyster mortality

Before the beginning of oyster mortality (on August 18), *Cyanobacteria* showed higher abundance at both the phylum and genus levels in July and August. In July, LEfSe analysis also identified significant enrichment of two dominant genera of *Cyanobacteria*, *Synechococcus_CC9902* and *Cyanoblum_PCC_6307. Cyanobacteria*, also known as blue–green algae, can form dense blooms, causing major problems in water quality such as increased turbidity (12) and oxygen depletion (25), which may induce hypoxia and anoxia, leading to the death of fish and benthic invertebrates. In addition, bloom-forming *Cyanobacteria* produce a variety of toxic secondary metabolites, including microcystins, nodularins, cylindrospermopsins, anatoxins, and saxitoxins, which can harm marine organisms (26). Thus, the tremendous increase in *Cyanobacteria* abundance had a negative impact on oyster survival.

The relationship between the biomarker genera and environmental factors showed that the abundances of *Synechococcus_CC9902* and *Cyanoblum_PCC_6307* were positively correlated with water temperature (Fig. 5C), indicating that the increase in *Cyanobacteria* was driven by rising temperature rather than randomness. This is robustly supported by an analysis of 11 years of satellite data from Lake Taihu, a large, shallow lake in the Yangtze River Delta in China, which showed that high temperatures and nutrient concentrations in spring promote the growth of *Cyanobacteria* (27). In severe cases, *Cyanobacterial* blooms covered the entire surface area of ~2,400 km^2^ (6). Many bloom-forming *Cyanobacteria* reach their maximal growth rates at relatively high temperatures, often above 25°C (6). During the July—August period in this study, the water temperature ranged from 23.0°C to 27.9°C, with an average value of 25.4°C (23), which was suitable for the massive proliferation of *Cyanobacteria*. Our previous study demonstrated that rising temperatures significantly altered the intestinal microbiota of oysters, promoted the proliferation of potential pathogens, and disrupted the immune status of susceptible oysters (23). Building upon these findings, we believe that elevated temperatures exert a multifaceted effect on host immune functions, pathogen load, and the surrounding microbial community ecology, further validating our prior conclusion that rising temperatures trigger oyster mortality.

### Environmental factors significantly affected the water microbial structure and composition

The results of ANOVA and RDA proved that water temperature and dissolved oxygen had a significant impact on the structure and composition of the water microbiota, accounting for each group clustered together independently depending on the month, with little overlap among groups. VPA quantitatively revealed that water temperature and dissolved oxygen accounted for 50.4% and 19.3% of microbial variations, respectively. Microbial communities are diverse, with some being aerobic and some anerobic; some prefer high temperatures, while some prefer cold temperatures (28). Therefore, water temperature and dissolved oxygen that drive the microbial community are ubiquitous in many ecosystems, such as mangrove ecosystem (29), core reef ecosystem (30), and aquatic ecosystem (21). Moreover, our previous investigation found that water temperature significantly shapes the intestinal microbiota of oysters, and resistant oysters maintain an enhanced capacity to regulate their symbiotics (23). Unlike the monthly clustering of water microbiota, gut microbial samples from oysters gathered into two clusters of Jul–Aug cluster and Jun–Oct–Nov cluster, and samples from corresponding months were dispersed irregularly within these two clusters (23). This comparable pattern indirectly indicates that host factors (deterministic processes) significantly affect microbiota assembly in oysters.

The relationship between environmental factors and the abundance of genera also revealed seasonal variations in the water microbiota. Among the genera that were significantly associated with environmental factors, 82% were significantly correlated with water temperature and dissolved oxygen. Nine of the 12 biomarker genera showed significant correlations with the water temperature or dissolved oxygen. In addition to *Cyanobium_PCC_6307* and *Synechococcus_CC9902* discussed previously, several other genera associated with nutrient utilization and phytoplankton proliferation were identified during colder months. For instance, *NS4_marine_group* is strongly correlated with inorganic nutrients such as ammonium and phosphate and is utilized as an indicator of increased phytoplankton concentration (31). *Marivita*, which is subordinate to *Verrucomicrobia*, can reduce nitrate to ammonium for microalgae growth (32). *Lentibacter*, another *Verrucomicrobia* member, has been repeatedly isolated from algae-rich waters at various geographical locations (33). These results suggest that phytoplankton is abundant in Rushan waters during the colder months, which is beneficial for the growth and fattening of cultured oysters. This may be one of the important factors contributing to the superior quality of “Rushan oyster.”

### Marked divergence in the microbial composition and functions between oysters and ambient water

The comparison of microbial composition between oysters and water demonstrated that ≤21% of the genera were shared between the two, and 50% of the identified genera were exclusively detected in oysters, suggesting that the oyster intestinal microbiota was predominantly composed of resident microbes rather than being a reflection of their surroundings. Similar results were reported in an investigation of *L. vannamei*, which found that the composition and abundance of dominant genera differed distinctly among the water, sediment, and intestines in shrimp ponds, regardless of rearing time (18). Zheng *et al.* (20) also discovered statistically significant differences at the phylum and genus levels between the microbiota composition in the intestine of *Pinctada fucata martensii* and surrounding water. This means that the microbiota in the water cannot easily transfer into oysters. Furthermore, variation in the abundance of shared genera differed between the oysters and their surroundings. Several common pathogens, such as *Vibrio*, *Coxiella*, and some genera in *Proteobacteria*, were less abundant in oysters, whereas *Firmicutes*, which are known to promote efficient dietary energy harvesting by the host through carbohydrate fermentation and lipid intake (34), were more enriched in oysters than in water, indicating that oysters might have the capacity to select beneficial microbiota and maintain some barrier mechanisms to prevent the entry of unfavorable bacteria. An investigation of *C. virginica* conducted by Stevick *et al*. (21) revealed that only 135 (45%) orders were shared between water and oyster guts and 161 (53%) were found exclusively in the oyster guts, suggesting that oysters selected and amplified rare members from water, while retaining the majority of members previously determined by host factors. Hence, the majority of the microbiota in oysters are resident members governed by deterministic factors, and only a small portion of the microbial communities are transients and driven by a stochastic process (11).

The selectivity of the oysters for the microbiota was further confirmed by the predicted functions of the shared genera. Consistent with the microbiota composition pattern, the functions of the shared genera differed significantly between oysters and water. This is not uncommon because enriched functions are predicted based on the divergent copy numbers of OTUs using the PICRUSt2 algorithm (35). However, the opposite effects were detected in oysters and water. Catabolic functions associated with pathways such as fucose, rhamnose, lactose, and galactose degradation were enriched in oysters. These polysaccharides have diverse biological activities but are difficult to degrade by host digestive enzymes (36). Nonetheless, non-digestible polysaccharides can be utilized by the intestinal flora to regulate metabolite production and the composition of the intestinal flora, further affecting the immune system, preventing pathogens from adhering to the intestinal surface, and protecting the intestinal epithelial barrier (36, 37). The enriched catabolic functions indicated that the intestinal flora of the oysters promoted the digestion of non-digestible nutrients. Some synthetic pathways such as adenosylcobalamin and L-tryptophan biosynthesis have also been detected in oysters. Adenosylcobalamin, the active form of vitamin B12, cannot be endogenously synthesized in animals, and its deficiency disturbs carbohydrate, fat, and amino acid metabolism (38). Tryptophan, an essential amino acid in all animals, is synthesized and provided at higher trophic levels by bacteria, fungi, and plants (39). Thus, microorganisms play an important role in supplying essential nutrients to oysters, even though oysters may obtain these substances from phytoplankton. Conversely, in water, the enriched pathways were mainly involved in assimilation and biosynthesis functions, such as chlorophyllide biosynthesis and glyoxylate assimilation. Chlorophyllide a is essential for the growth and survival of photosynthetic bacteria, like *Cyanobacteria* (26). The glyoxylate cycle allows bacteria to use two-carbon compounds as carbon sources for growth and energy production, which is particularly important for bacteria living in nutrient-poor environments, such as soil and water (40). The pattern detected in the water suggested that the functions of the water microbiota mainly revolved around maintaining essential life processes for their own growth and survival. Therefore, based on the differences in composition and functions between oyster and water microbiota, oysters may select probiotics and deny unfavorable bacteria, which further supports the argument that deterministic factors govern the assembly of oyster intestinal microbiota.

### Conclusion

**Fig 8:**
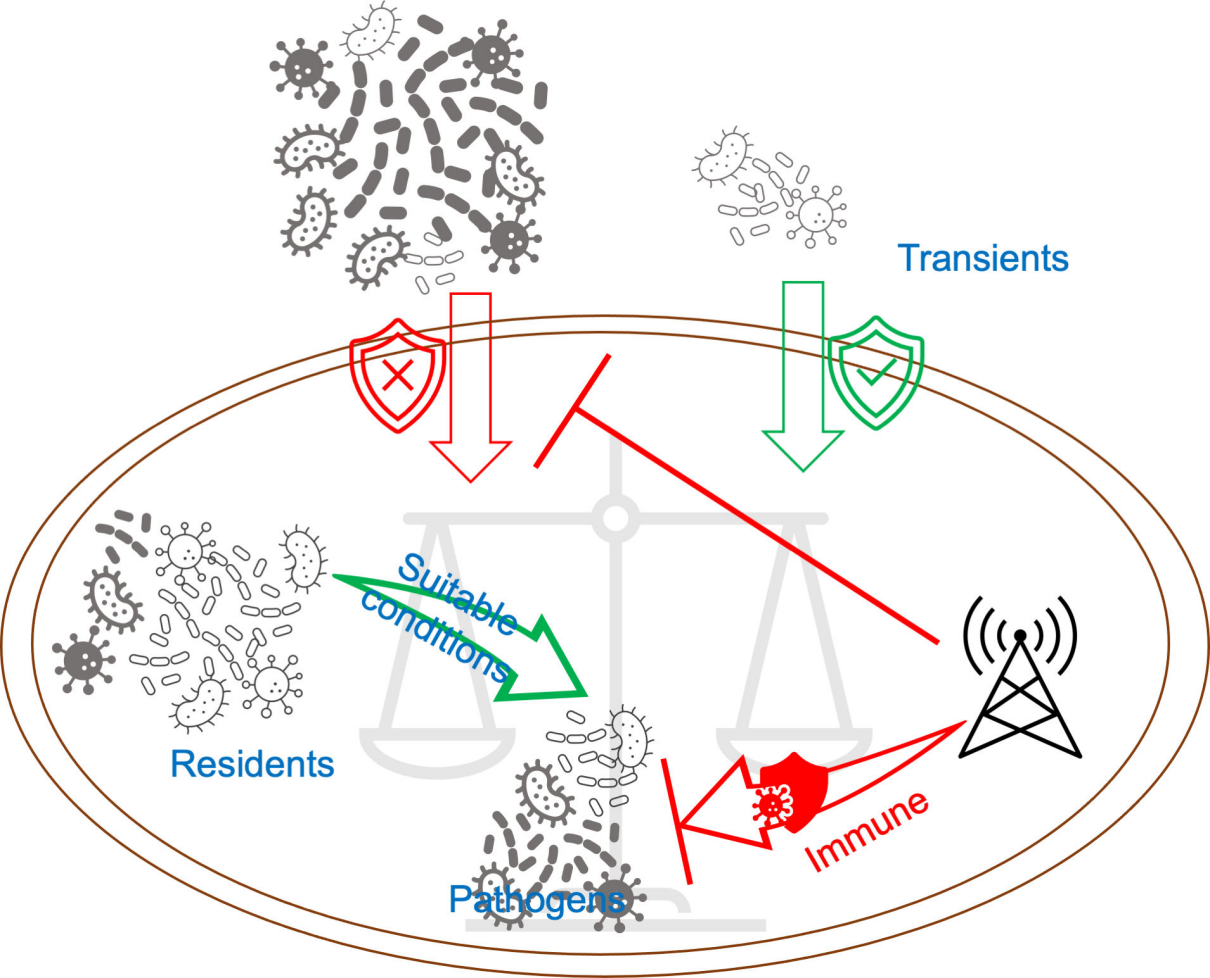
Microbiota plasticity of oysters. Microbiota within oysters comprises two main components. The majority consists of resident members, encompassing both beneficial probiotics and pathogenic species. Under favorable conditions, the pathogenic residents can multiply to a threshold level, triggering the oyster’s immune response mechanisms. The remaining portion consists of transient microbes from the surrounding environment, which are regulated by the oyster’s selective or barrier mechanisms to prevent unrestricted microbial access to their bodies. In the ongoing battle between defense mechanisms and pathogen proliferation, the surrendering of oysters increases the risk of mortality.

Building on our previous work (23) exploring the connection between hosts and pathogens, this study examined the relationship between the microbiota in the aquatic environment and resident oysters during an oyster mortality event. From an ecological perspective, the outbreak of *Cyanobacteria* in July and August may have promoted oyster mortality. Taking into account our previous findings (23), we speculated that the microbiota in the oyster intestine is composed of two components. A small proportion of transients penetrate from the water into the oyster, controlled by stochastic processes, which might be nonfatal, as oysters maintain selective or barrier mechanisms governed by deterministic factors to inhibit the free movement of microbes into their bodies. The majority are residents, regulated by the host and comprising both probiotics and pathogens. In healthy oysters, or under conditions unfavorable for pathogen proliferation, pathogens are maintained at low levels and have less impact on the growth and survival of oysters. Once conditions such as rising temperatures become suitable, pathogens can multiply rapidly. When the load of pathogens rises above a certain threshold, the oyster can activate their immunological functions to inhibit further proliferation of pathogens. This process is also governed by determinative factors. If the immune response is successful, the oyster returns to its normal physiological state and survives; if it fails, the oyster dies. In conclusion, the microbial communities in the oyster intestine exhibit some plasticity manifested in 1) the oyster’s selective or barrier mechanism, admitting a small portion of inoffensive external transients, and 2) internal immune mechanisms countering unfavorable bacterial proliferation (Fig. 8). The deterministic process dominates the microbiota assembly of oysters, regardless of whether oyster mortality occurs. This study may unveil a common mechanism by which marine invertebrates adapt to complex microbial surroundings under diverse environments.

## MATERIALS AND METHODS

### Water sample collection and environmental factor monitoring

Water samples were collected synchronously with oysters from an oyster farm in 2020 in Rushan, China, as described in our previous study (23), and labeled as W06 (June 23), W07 (July 30), W08 (August 18), W09 (September 22), W10 (October 28), and W11 (November 28). At each date, three bottles of 500 mL water were collected near the oyster cages and filtered separately *in situ* by using a polycarbonate membrane with a pore size of 0.22 µm. Following filtration, the membrane was placed in a 2.0-mL sterile centrifuge tube, frozen in liquid nitrogen, and then stored at −80°C. Another 500 mL water was collected into a blackout bottle and filtered *in situ* using a 0.45-µm polycarbonate membrane, and chlorophyll a (Chl) was then tested according to the standard method (41). Other environmental factors, including temperature (T), dissolved oxygen (Do), and salinity (Sal), were the same as those in previous studies (23).

### DNA extraction and 16S rRNA amplicon sequencing

Microbial DNA was extracted from the filtered membranes at each sample point using HiPure Soil DNA Kits (Magen, Guangzhou, China), following the manufacturer’s protocols. The quality of the extracted DNA was determined by agarose gel electrophoresis, and the DNA concentration was measured using a NanoDrop spectrophotometer (Thermo Fisher Scientific, USA). High-throughput sequencing of the 16S rRNA V3–V4 region was performed using the Illumina NovaSeq 6000 by Gene Denovo Biotechnology Co. (Guangzhou, China).

### Amplicon sequencing data analysis

Data filtering, OTUs and taxonomy classifier, and abundance and diversity calculations were performed as previously described (23). The influence of environmental factors on each index of α diversity was tested by multifactor analysis of variance (ANOVA) using the “agridat” package (42) in the R statistical environment. Principal coordinate analysis (PCoA) and analysis of similarity (Adonis and Anosim) were performed to analyze the dissimilarity in the microflora structure based on the Bray—Curtis distance. Liner discriminant analysis (LDA) effect size analysis (LEfSe) was conducted to screen for biomarkers that were statistically different between various groups with an LDA score >4 (43). Redundancy analysis (RDA) and variation partition analysis (VPA) were executed in the R project “Vegan” package (44) from the OTU and genus levels. Pearson’s correlation between environmental factors and screened biomarkers was calculated in the R project “psych” package (45) and visualized in Cytoscape (version 3.9.1) (46). To mitigate potential inaccuracies arising from different sequencing batches, the raw data of water samples, susceptible oysters, and resistant oysters were re-analyzed via EasyAmplicon (47). Venn analysis between groups of water, susceptible oysters, and resistant oysters was performed in the R project “VennDiagram” to determine the shared taxonomies between them (48). The abundance heatmap of OTUs and shared genera between water and oyster was generated using TBtools 1.118 (49), where the Euclidean distance and average cluster method were set to determine the clustering patterns. PICRUSt2 (https://github.com/ picrust/picrust2/) was used to predict the functional profiling of shared genera in water and oysters, and the difference between them was evaluated using STAMP 2.1.3 (50).

### Statistical analysis

Statistical analyses were conducted using R software (https://www.r-project.org) and related packages. Data visualization was performed using R (https://www.r-project.org), TBtools 1.118 (49), and GraphPad Prism 9 for MacOS (GraphPad Software, San Diego, California, USA, www.graphpad.com). The level of significance was set at *P* < 0.05.

## Data Availability

The raw sequencing data of this study have been deposited in the NCBI under BioProject PRJNA869216 (SRA accession No.: SRR25236528~SRR25236545).

## SUPPLEMENTAL MATERIAL

Figure S1 spectrum.02881-23-s0001.eps Redundancy analysis (RDA).

Figure S2 spectrum.02881-23-s0002.eps Relative abundance of top 10 phyla of microbiota in water.

Table S1 spectrum.02881-23-s0003.xls Data assessment.

Table S2 spectrum.02881-23-s0004.xls Environmental factors.

ASM does not own the copyrights to Supplemental Material that may be linked to, or accessed through, an article. The authors have granted ASM a non-exclusive, world-wide license to publish the Supplemental Material files. Please contact the corresponding author directly for reuse.
